# Cardiovascular responses to dynamic and static upper-body exercise in a cold environment in coronary artery disease patients

**DOI:** 10.1007/s00421-021-04826-x

**Published:** 2021-10-16

**Authors:** Rasmus I. P. Valtonen, Heidi H. E. Hintsala, Antti Kiviniemi, Tuomas Kenttä, Craig Crandall, Wouter van Marken Lichtenbelt, Juha Perkiömäki, Arto Hautala, Jouni J. K. Jaakkola, Tiina M. Ikäheimo

**Affiliations:** 1 Center for Environmental and Respiratory Health Research (CERH), University of Oulu, 5000, 90014 Oulu, Finland; 2 Medical Research Center, University of Oulu, Oulu University Hospital, Oulu, Finland; 3 Centria University of Applied Sciences, Kokkola, Finland; 4 Department of Internal Medicine, University of Texas Southwestern Medical Center, Institute for Exercise and Environmental Medicine, Texas Health Presbyterian Hospital, Dallas, USA; 5 Department of Nutrition and Movement Sciences, NUTRIM School of Nutrition and Translational Research in Metabolism, Maastricht University, Maastricht, The Netherlands; 6 Research Unit of Internal Medicine, Medical Research Center Oulu, University of Oulu, Oulu University Hospital, Oulu, Finland; 7 Faculty of Sport and Health Sciences, University of Jyväskylä, Jyväskylä, Finland; 8 Department of Community Medicine, University of Tromsø, Tromsø, Norway

**Keywords:** Cold, Exercise, Upper body, Dynamic, Static, Coronary artery disease

## Abstract

**Purpose:**

Upper-body exercise performed in a cold environment may increase cardiovascular strain, which could be detrimental to patients with coronary artery disease (CAD). This study compared cardiovascular responses of CAD patients during graded upper-body dynamic and static exercise in cold and neutral environments.

**Methods:**

20 patients with stable CAD performed 30 min of progressive dynamic (light, moderate, and heavy rating of perceived exertion) and static (10, 15, 20, 25 and 30% of maximal voluntary contraction) upper body exercise in cold (− 15 °C) and neutral (+ 22 °C) environments. Heart rate (HR), blood pressure (BP) and electrocardiographic (ECG) responses were recorded and rate pressure product (RPP) calculated.

**Results:**

Dynamic-graded upper-body exercise in the cold increased HR by 2.3–4.8% (*p* = 0.002–0.040), MAP by 3.9–5.9% (*p* = 0.038–0.454) and RPP by 18.1–24.4% (*p* = 0.002–0.020) when compared to the neutral environment. Static graded upper-body exercise in the cold resulted in higher MAP (6.3–9.1%; *p* = 0.000–0.014), lower HR (4.1–7.2%; *p* = 0.009–0.033), but unaltered RPP compared to a neutral environment. Heavy dynamic exercise resulted in ST depression that was not related to temperature. Otherwise, ECG was largely unaltered during exercise in either thermal condition.

**Conclusions:**

Dynamic- and static-graded upper-body exercise in the cold involves higher cardiovascular strain compared with a neutral environment among patients with stable CAD. However, no marked changes in electric cardiac function were observed. The results support the use of upper-body exercise in the cold in patients with stable CAD.

**Trial registration:**

Clinical trial registration NCT02855905 August 2016.

## Introduction

It is well established that the cold season is associated with increased morbidity and mortality, which is often cardiovascular related (Sun et al. 2018; Liu et al. 2015; Fares 2013). Cardiac workload is higher in the cold, due to increased peripheral vascular resistance and related elevated blood pressure (Castellani and Young 2016). Adding exercise to cold exposure may increase cardiovascular strain further (Ikäheimo 2018; Manou-Staphopoulou et al. 2015), given an increased incidence of myocardial infarctions related to winter sports (Klug et al. 2011) or heavy exercise, such as snow shoveling (Nichols et al. 2012; Janardhanan et al. 2010). Importantly, these cardiovascular events are more common among populations with ischemic heart disease (Toukola et al. 2015). The reason for these events could be related to the high myocardial oxygen demand caused by the combined effects of cold exposure and exercise that cannot be met by a myocardial blood flow-limiting disease, such as coronary artery disease (CAD) (Ikäheimo 2018; Manou-Staphopoulou et al. 2015). Our previous study with CAD patients showed that lower-body aerobic exercise is associated with a 20% higher cardiac workload in a cold compared with neutral environment (Valtonen et al. 2018). The mismatch between myocardial demand and blood flow during exercise in the cold may result in earlier appearance of myocardial ischemia (Meyer et al. 2010).

Upper-body exercise performed in a cold environment, such as wood chopping, snow shoveling, and skiing can be particularly strenuous for the cardiovascular system. First, upper-body exercise itself is carried out with a relatively small muscle mass, and which increases arterial BP and overall cardiovascular strain considerably (Calbet et al. 2015; Miles et al. 1989). Second, the effect on hemodynamics differ according to the exercise mode, e.g., between static and dynamic exercise. Dynamic aerobic exercise involves cycles of muscular contraction and relaxation and where perfusion increases considerably during the relaxation phase. In contrast, static upper-body exercise causes mechanical compression of muscles and the vasculature, as well as pressure loading (Osada et al. 2015; Tanaka et al. 2014). This pressure load may increase the risk of myocardial ischemia particularly among CAD patients (Manou-Staphopoulou et al. 2015). Finally, cold-induced changes in circulation and cardiac function could further influence upper-body exercise hemodynamics, but such information is lacking.

The aim of this study was to examine cardiovascular responses during upper-body dynamic and static exercise in a cold environment. We tested the hypothesis that cardiac workload is higher during dynamic and static upper-body exercise in cold compared to a neutral environment. We further assumed that signs of myocardial ischemia would be observed during exercise in the cold, and especially at a higher exercise intensity. We investigated CAD patients, representing a risk population that experiences adverse cardiovascular health events in the winter.

## Methods

### Patients

Oulu University Hospital patients [*n* = 20, aged 59.4 ± 8.8 years, height: 173.9 ± 5.3 cm, weight 84.5 ± 14.1 kg, BMI: 27.9 ± 3.9 kg/m^2^ (mean ± SD)] were recruited (Table 1). We selected a total of 20 participants, with that number being based on a sample size estimation and power analysis that indicated that statistically significant differences in BP between cold exposure and baseline [Power (1-ß err prob), 0.9, Cohen’s effect size 0.8, *α* err prob 0.05] would be expected with just 15 participants. The inclusion criteria consisted of a diagnosed CAD [Canadian Cardiac Society (CCS) class I–II] and a non-ST-elevation myocardial infarction at least 3 months prior to experimentation. The exclusion criteria were: CCS class III–IV, previous myocardial infarction less than 3 months prior to experimentation, chronic atrial fibrillation, claudication, unstable angina pectoris, left ventricular ejection fraction less than 40%, a history of coronary artery bypass grafting, pacemaker, serious complex or ECG anomalies during rest, asthma or diabetes and current smoking. An experienced cardiologist evaluated the inclusion and exclusion of each subject based on the criteria defined above. The participants received both oral and written information of the study and a signed informed consent was required for participation. The study as approved by the Ethics Committee of Oulu University Hospital District. The study is registered in the Clinical Trials (NCT02855905).

**Table 1:** Characteristics of the study population of CAD patients (*n* = 20)

Variables	Mean (SD) or *n* (%)
Age, years	59 (8.5)
Body mass index, kg/m^2^	28 (3.9)
Body fat, %	24 (6.0)
Peak oxygen consumption, mL/kg/min	31 (5.5)
Systolic blood pressure, mmHg	120 (13)
Diastolic blood pressure, mmHg	77 (11)
Hypertension, n	18 (90%)
Time from MI, months	29 (11)
Single vessel disease, *n*	13 (65%)
Double vessel disease, *n*	5 (25%)
Triple vessel disease, *n*	2 (10%)
Number of stents, *n*	2 (0–4)
Left ventricular ejection fraction	63% (8.5)
Medication	
Acetylsalicylic acid, *n*	20 (100%)
Beta-blockers, *n*	15 (75%)
Statins, *n*	16 (80%)
Angiotensin converting enzyme inhibitors, *n*	8 (40%)
Angiotensin receptor blockers, *n*	6 (30%)
Calcium channel blockers, *n*	3 (15%)
Pensioner, *n*	12 (60%)
Perceived health, *n*	
Excellent	4 (20%)
Quite good	8 (40%)
Average	7 (35%)
Quite poor/very poor	1 (5%)
Any use of alcohol	19 (95%)
Physical demands of work, *n*	
Mainly sitting	13 (65%)
Much walking	2 (10%)
Much waking and lifting	5 (25%)
Heavy manual labor	0 (0%)
Leisure-time physical activity, *n*	
Never	1 (5%)
Rarely	12 (60%)
Often	5 (25%)
Very often	1 (5%)
Physical fitness status, *n*	
Excellent	3 (15%)
Quite good	9 (45%)
Average	7 (35%)
Quite poor	1 (5%)

Values are the number of the patients or means ± SD. Peak oxygen consumption, in mL/kg/min, was estimated (3.5*MET, where MET is metabolic equivalent of task) from a symptom-limited maximal oxygen uptake test

Clinical exercise tests were performed approximately a month prior to the experiments to assess maximal exercise capacity and to detect possible ECG abnormalities, indicating cardiac ischemia, during a graded cycle ergometer test (Ergoline, ergoselect 100 K, Fysioline, Finland). Prior to the test, ECG and HR were measured at rest in the supine position. The test was started from at 30 W and was increased by 15 W each minute until exhaustion. An exercise physiologist carried out the tests, which were monitored by a medical doctor. No abnormalities were detected in the ECGs during the exercise test in any of the enrolled subjects.

Each patient took part in the following four experimental conditions, administered in random order: (1) dynamic upper-body exercise in a cold (− 15 °C) and (2) neutral (+ 22 °C) environment, as well as (3) static upper-body exercise in a cold (− 15 °C) and (4) neutral (+ 22 °C) environment. Dynamic upper-body exercise consisted of 5-min pre-exposure rest, three 5-min work cycles via an arm crank ergometer (Monark 881E, Vansbro, Sweden), each with a different intensity, and two 4-min rest periods between the exercise bouts. The pedaling speed was adjusted prior to the experiments and was based on subjective judgements of perceived exertion of mild (11–12 fairly light) moderate (13–14 somewhat hard) and high (15–16 hard) intensities (Borg 1998). The same speed was applied in the cold and neutral environments. The level of static upper-body exercise was adjusted based on maximal bench press voluntary contraction (MVC) [Newtest Leg Force (bench press mode), Newtest, Oulu, Finland]. MVC was measured in the beginning of the first visit to the lab and at least 1-h before the baseline measurements. The exercise itself consisted of 5-min pre-exposure resting and then five 1.5-min isometric contractions at the following workloads: 10, 15, 20, 25 and 30% of MVC. Patients had a 4-min break following each work cycle. The patients were instructed to avoid heavy exercise 24 h before and alcohol 48 h before and coffee/caffeine related beverages 2 h prior to the experiments. Prior to initiating the experiments, body composition (e.g., fat %) was assessed from each subject by bioimpedance measurements (InBody720 Biospace, Seoul, Korea). Subjects also completed a questionnaire related to health and lifestyle and inquired about medication, alcohol consumption, physical fitness and current health status.

Brachial blood pressure (BP) (Schiller BP 200 + , Switzerland) was assessed at 5 min intervals during baseline and follow-up. Throughout the trials, BP was measured before and immediately following each exercise bout (and not while exercising). The subjects were instructed to set their arm in a stable position on the table immediately after the work bout. RPP was calculated by multiplying brachial systolic BP with HR. Physical strain was evaluated objectively by HR and subjectively by Borg’s perceived of exertion scale (Borg 1998). HR was monitored continuously, and perceived exertion was obtained at 5 min intervals throughout the intervention.

ECG was recorded and monitored continuously using a 15-lead ECG (Cardiosoft V6.71, GE Healthcare, Freiburg, Germany). The placements of the ECG electrodes at rest followed the standard 12 lead placement and X, Y, Z leads. In the clinical exercise test and during the interventions, the arm and foot electrode were reset to both shoulders and lower back. Signal analyses were carried out with custom-made software in Matlab (MathWorks, inc., Natic, MA, USA). The software detected ectopic and abnormally shaped beats and removed them from the analysis. For each ECG lead, representative beats from ten consecutive beats were formed throughout the recording. These beats were then automatically analyzed for QRS duration, QT interval and R- and T-wave amplitudes. The interval and amplitude measurements were visually verified and manually adjusted if needed. Furthermore, the QT interval was corrected with the nomogram method (QTc) (Karjalainen et al. 1994).

While being exposed to cold, the patients wore full winter clothing consisting of underwear (shirt, pants), insulated trousers, insulated jacket, overtrousers, overjacket, socks and shoes (insulation value of clothing ensemble 2.13 clo). This clothing ensemble was selected to replicate the clothing one would wear in the winter months during physical exertion such as snow shoveling. A lesser amount of insulation (0.75 clo) was used at neutral climate exposures to avoid heat strain.

Skin temperature was measured continuously using thermistors (NTC DC95, Digi-Key, Thief River Falls, MN, USA) attached to the right scapula, left cheek, forehead, left calf, right anterior thigh, dorsal side of left index finger (middle phalanx), left hand, left forearm, right shoulder, left upper chest. Data were recorded at 20 s intervals with two temperature data loggers (SmartReaderPlus; Acr Systems Inc., BC, Canada). Mean skin temperature (Tsk) was calculated as follows: ${\text{tsk}} = \sum {\text{k}}_{{\text{i}}} \times {\text{tsk}}_{{\text{i}}} = \left[ {0.0{7} \times {\text{forehead}} + 0.{175} \times {\text{right scapula}} + 0.{175} \times {\text{left upper chest}} + 0.0{7} \times {\text{right arm}} + 0.0{7} \times {\text{left arm}} + 0.0{5} \times {\text{left hand}} + 0.{19} \times {\text{right anterior thigh}} + 0.{2} \times {\text{left calf}}} \right].$ (ISO 9886). Thermal sensations were inquired using scales of perceptual judgements on personal thermal state (ISO 10551).

### Statistical analyses

We conducted two-way ANOVAs separately for static and dynamic upper body exercise where the main effects of temperature (cold vs. neutral) and time (baseline vs. intervention) were compared. For any observed interaction, separate post hoc analyses (Bonferroni’s tests considering multiple comparisons) were carried out to compare means between the temperature conditions. The results are expressed as means and their standard deviations (SD). Statistical significance was set at *p* < 0.05. Statistical analyses were performed with IBM SPSS for Windows version 23 (IBM Corp, Armonk, NY, USA).

## Results

The characteristics of the participants are presented in Table 1.

### Dynamic exercise

**Fig. 1:**
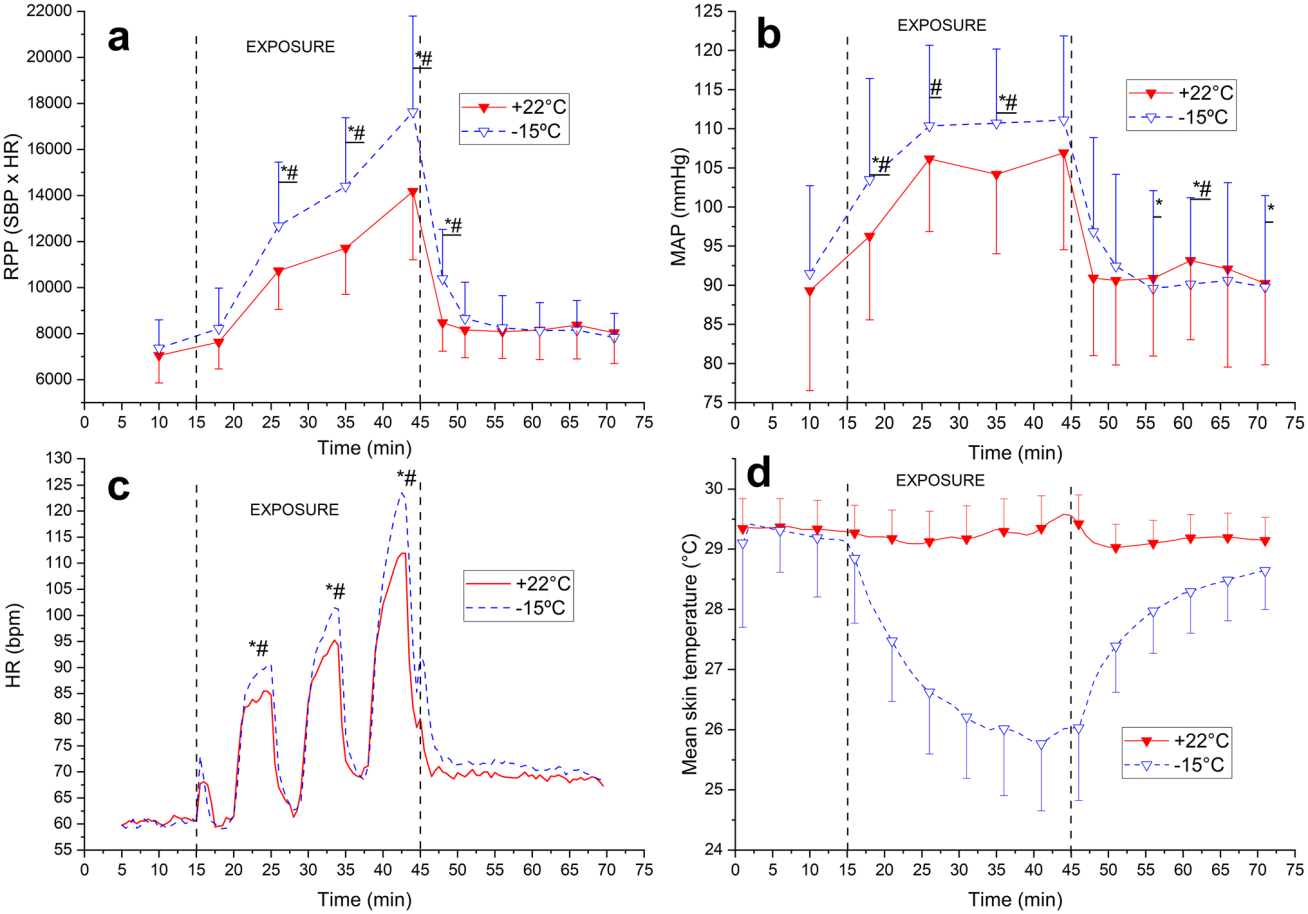
Rate pressure product (RPP), Mean arterial blood pressure (MAP), heart rate (HR), and mean skin temperature (T_sk_) during dynamic upper-body exercise at + 22 °C and − 15 °C (*n* = 20). The vertical dotted lines represent the start and end of the exercise intervention. Values represent means and their SD. Significantly different from * baseline and # exercise at + 22 °C (*p* < 0.05)

Exposure to cold decreased T_sk_ by 3.7 °C (*p* < 0.001) and facial skin temperature decreased considerably from + 31 °C to + 15 °C (*p* < 0.001) during dynamic exercise in the cold environment (Fig. 1). At the end of the intervention, the average whole-body thermal sensation of patients was − 1/slightly cool (cold dynamic) and + 2/warm (neutral dynamic). The identical pedaling speeds represented 56, 62 and 73% of HR_max_ in a neutral and 59, 66 and 80% of HR_max_in a cold environment, with HR_max_ values derived from the incremental leg exercise test. The RPE during dynamic exercise varied from fairly light to hard (11–15) at the neutral temperature and from somewhat hard to very hard (12–16) in the cold environment. T_sk_ remained at a lowered level throughout the follow-up period after exercise in the cold compared with a neutral environment (Fig. 1).

When compared to neutral environmental conditions, dynamic exercise in the cold increased HR by 2.3–4.8% (*p* = 0.002–0.040), MAP by 3.9–5.9% (*p* = 0.038–0.454) and RPP by 18.1–24.4% (*p* = 0.002–0.020). The recovery of RPP following dynamic exercise in a cold environment was delayed and reached the same level as the neutral exercise intervention 10 min after the end of the exposure. (Fig. 1).

Recorded and calculated ECG parameters during dynamic upper-body exercise in + 22 and − 15 °C are presented in Table 2. QT interval was shorter at all levels of dynamic exercise in the cold compared to neutral environment (*p* < 0.020). 60% of the patients demonstrated ST-segment depression exceeding 1 mm (channels II, V2–V5) from baseline during the last dynamic work cycle regardless of the thermal environment (11 and 12 of the 19 patients, correspondingly). Maximum ST-segment depression was 1.2 mm in the neutral environment and 1.5 mm in the cold.

**Table 2:** Electrocardiographic responses of CAD patients (*n* = 20) during dynamic upper body exercise of different intensities [light (RPE 11–12), moderate (RPE 13–14) and hard (RPE 15–16)] in a neutral (+ 22 °C) and cold (− 15 °C) environment

	Baseline	Light	Moderate	Hard
+ 22 °C				
HR	60 ± 6.7	83 ± 8.5*^#^	92 ± 10.7*^#^	108 ± 12.9*^#^
QRS	96 ± 10.1	97 ± 9.5	97 ± 8.6	98 ± 9
QT	422 ± 14.3	381 ± 15.6*^#^	365 ± 15.7*^#^	345 ± 19.9*^#^
QTc	423 ± 13.1	423 ± 13.6	456 ± 49.1	480 ± 52.2
R-amp	3.4 ± 1	3.5 ± 1	3.6 ± 1	3.5 ± 1
T-amp	1.2 ± 0.4	1.2 ± 0.4	1.1 ± 0.4	1.1 ± 0.3
− 15 °C				
HR	60 ± 6	89 ± 8*^#^	100 ± 11.3*^#^	121 ± 15.2*^#^
QRS	96 ± 10	98 ± 8	97 ± 11.2	98 ± 10.6
QT	422 ± 14	369 ± 20*^#^	351 ± 17.5*^#^	319 ± 26.1*^#^
QTc	422 ± 13	426 ± 25	454 ± 57.5	498 ± 33.1
R-amp	3.5 ± 1	3.6 ± 1.1	3.6 ± 1.1	3.5 ± 1.1
T-amp	1.3 ± 0.4	1.2 ± 0.3	1.2 ± 0.3	1.2 ± 0.3

*HR* heart rate, *QRS* Duration of QRS, *QT* QT interval, *QTc* corrected for HR by the nomogram method, *R-amp* R peak amplitude, *T-amp* T peak amplitude

*Significantly different from baseline (*p* < 0.05) and # exercise at + 22 °C (*p* < 0.05). Values represent group means ± SD

### Static exercise

**Fig. 2:**
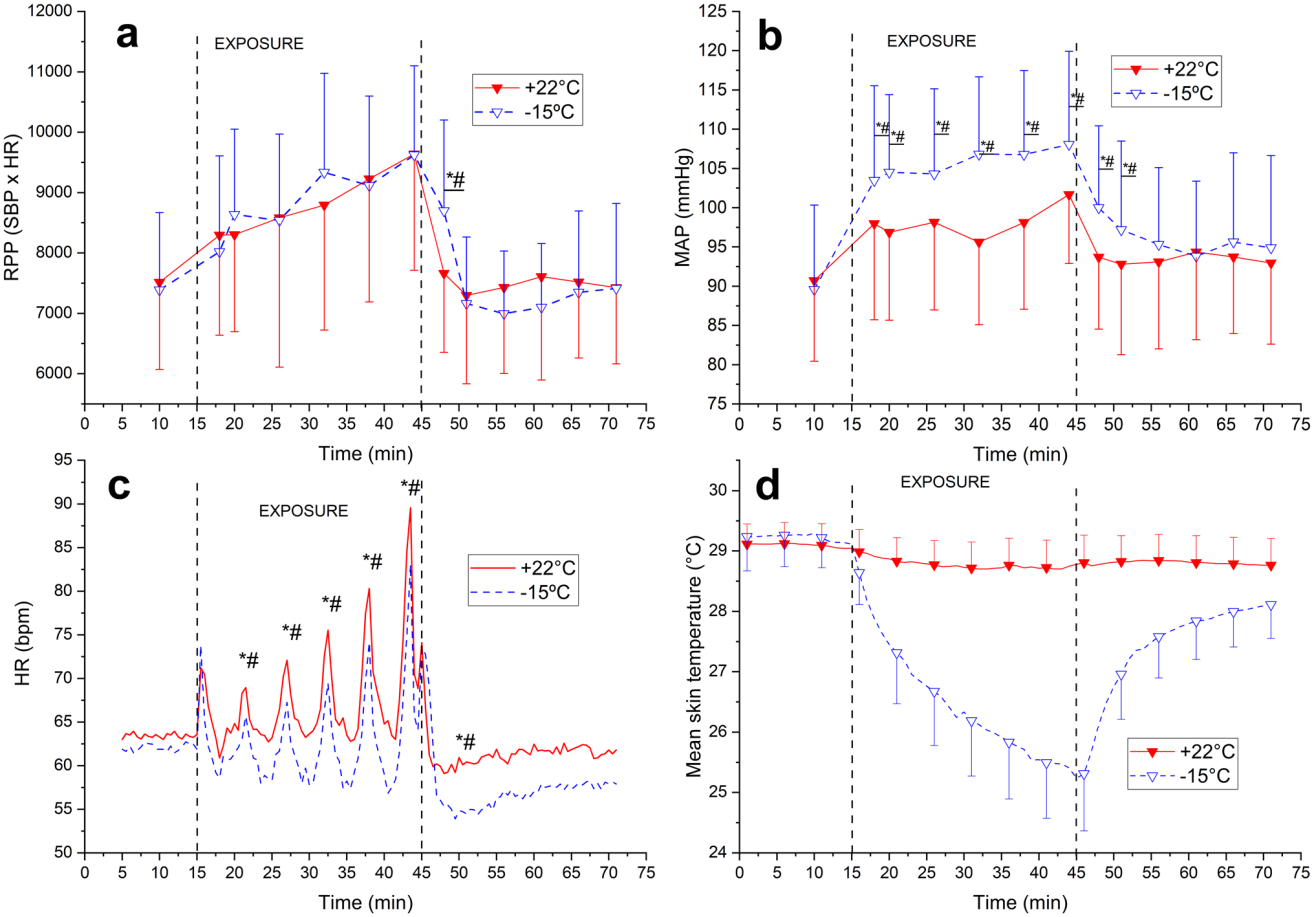
Rate pressure product (RPP), Mean arterial blood pressure (MAP), heart rate (HR), and mean skin temperature (T_sk_) during static upper-body exercise either at + 22 °C and − 15 °C (*n* = 20). The vertical dotted lines represent the start and end of the exercise intervention. Values represent means and their SD. Significantly different from * baseline and # exercise + 22 °C (*p* < 0.05)

Exposure to cold temperature decreased T_sk_ by 4.1 °C (*p* < 0.001) and facial skin temperature from + 31 to + 15 °C (*p* < 0.001) by the end of exercise in the cold environment (Fig. 2). At the end of the intervention, the average whole-body thermal sensation of patients was − 2/cold (cold) and + 1/slightly warm (neutral). The identical graded workloads represented 46, 47, 48, 52, and 56% of HR_max_ at neutral environment and 42, 43, 44, 47 and 50% in a cold environment, with HR_max_ values derived from the incremental leg exercise test. The RPE varied from fairly light to very hard (10–16) for both temperatures. T_sk_ remained at a lowered level throughout the follow-up period after exercise in the cold compared with a neutral environment (Fig. 2D).

Static exercise in the cold resulted in higher MAP (6.3–9.1%; *p* = 0.000–0.014), lower HR (4.1–7.2%; *p* = 0.009–0.033), but unaltered RPP compared to a neutral environment. The recovery of RPP following static exercise in the cold was delayed and reached the same level as the neutral exercise intervention 10 min after the end of the exposure (Fig. 2).

The ECG responses to graded static exercise are presented in Table 3. We did not observe any effects of environmental temperature on ECG parameters at any of the exercise intensities. In addition, the patients did not demonstrate ST-segment depression at any static exercise intensity.

**Table 3:** Electrocardiographic responses of CAD patients (*n* = 20) during static upper body exercise at different intensities (10–30% of MVC) in a neutral (+ 22 °C) and cold (− 15 °C) environment

	Baseline	10% MVC	15% MVC	20% MVC	25% MVC	30% MVC
+ 22 °C						
HR	62 ± 6	67 ± 7*^#^	71 ± 7*^#^	74 ± 7*^#^	79 ± 7*^#^	89 ± 12*^#^
QRS	96 ± 13	96 ± 13	96 ± 14	95 ± 14	96 ± 13	94 ± 15
QT	424 ± 20	414 ± 18	410 ± 19	409 ± 21	404 ± 26	396 ± 23
QTc	428 ± 17	430 ± 17	432 ± 14	437 ± 17	441 ± 21	466 ± 43
R-amp	3.4 ± 0.9	3.6 ± 0.8	3.6 ± 0.8	3.7 ± 0.8	3.8 ± 0.8	3.8 ± 0.9
T-amp	1.1 ± 0.5	1.2 ± 0.4	1.2 ± 0.4	1.2 ± 0.4	1.1 ± 0.4	1 ± 0.4
− 15 °C						
HR	62 ± 6	66 ± 7*^#^	67 ± 8*^#^	70 ± 9*^#^	74 ± 9*^#^	82 ± 12*^#^
QRS	97 ± 14	97 ± 14	96 ± 14	97 ± 13	97 ± 14	96 ± 13
QT	417 ± 21	414 ± 20	412 ± 21	411 ± 23	410 ± 19	402 ± 27
QTc	421 ± 16	427 ± 13	427 ± 17	431 ± 16	437 ± 14	441 ± 20^**#**^
R-amp	3.3 ± 0.9	3.6 ± 1	3.7 ± 0.9	3.7 ± 0.9	3.8 ± 0.9	3.8 ± 0.9
T-amp	1.1 ± 0.5	1.2 ± 0.4	1.2 ± 0.4	1.2 ± 0.4	1.2 ± 0.4	1.1 ± 0.4

*HR* heart rate, *QRS* Duration of QRS, *QT* QT interval, *QTc* corrected for HR according to the nomogram method, *R-amp* R peak amplitude, *T-amp* T peak amplitude

*Significantly different from baseline (*p* < 0.05) and # exercise at + 22 °C (*p* < 0.05). Values represent means ± SD for baseline and each exercise intensity

## Discussion

We demonstrate, for the first time, that both upper-body exercise in the cold involves higher cardiac workload (dynamic exercise) and augmented BP (static exercise) compared with a neutral environment among patients with stable CAD. This difference in cardiovascular strain remained consistent with increasing exercise intensity. Dynamic exercise in the cold was accompanied with higher BP, HR, and cardiac workload as assessed by RPP. In contrast, static exercise in the cold caused a higher BP, but lower HR and unaltered RPP. Despite of the higher cardiovascular strain during exercise in the cold, no marked changes in electric cardiac function were observed for either dynamic or static exercise, although ST depressions was detected for heavy dynamic upper-body exercise in both thermal conditions.

### Dynamic upper-body exercise in the cold

In accordance with our hypothesis, we detected 18–24% higher cardiac workload (RPP) during graded dynamic upper-body exercise in a cold compared with a neutral environment. The subjective ratings for the exercise intensities in the cold ranged from relatively light to hard and represented 59–80% of HR_max_. The observed higher RPP was related both to an increase in MAP and HR.

The observed higher cardiac workload during upper-body exercise in the cold resembles our previous study involving lower-body dynamic exercise of CAD patients (Valtonen et al. 2018), which can be explained by a few mechanisms. Dynamic exercise itself is related with increased blood flow to the working muscles, muscle and skin vasodilation, and related cardiac volume loading (Manou-Staphopoulou et al. 2015). As a result of upper body dynamic exercise, BP and HR increases, and consequently cardiac workload and myocardial oxygen demand also increases (Calbet et al. 2015; Miles et al. 1989). In addition, concurrent cold exposure, and associated cooling of the skin, results in peripheral and visceral vasoconstriction (Johnson et al. 2014; Charkoudian 2010; Wilson et al. 2007) and elevates MAP further (Castellani and Young 2016). Indeed, we observed a constantly decreasing T_sk_ also at the highest exercise intensities indicating whole-body superficial cooling. At the same time, an augmented HR while performing dynamic exercise in the cold may be related to higher sympathetic activity and withdrawal of parasympathetic activity to exercise itself (Gonzales-Camarena et al. 2000; Tulppo et al. 1999), coupled with further sympathetic activation related to cooling of the skin areas.

Previous studies examining cardiovascular responses to dynamic upper-body exercise in a cold environment are scarce. These studies have mainly examined the energy expenditure of habitual chores, such as snow shoveling among healthy persons (Franklin et al. 1995; Smolander et al. 1995) and CAD patients (Sheldahl et al. 1992) but did not separately examine the effect of a cold environmental temperature on cardiovascular responses. Upper-body dynamic exercise has also been examined in relation to double-poling performance during skiing in healthy athletes (Wiggen et al. 2016; Wiggen  et al. 2013), but these studies did not examine cardiovascular responses. Our recent study from the same data showed that dynamic upper-body exercise caused beneficial post-exercise BP lowering effect among CAD patients, regardless of the environmental temperature where the activity was carried out (Hintsala et al. 2021).

### Static upper body exercise in the cold

We hypothesized that a cold environment would increase cardiovascular strain during upper-body static exercise compared with the corresponding exercise in a neutral environment. Indeed, we observed 6–9% higher MAP during graded static exercise in cold. The subjective ratings of the various exercise intensities in the cold ranged from light to very hard and represented 44–50% of HR_max_. In contrast, HR remained significantly lower at all exercise intensities resulting in an unaltered cardiac workload compared with exercise in a neutral environment.

The mechanisms for the higher MAP during exercise in a cold environment could be due to a few factors. Static exercise itself is related with increased sympathetic activity (Machado-Vidotti et al. 2014), and a pressor response due to mechanical compression, reduced perfusion, accumulation of metabolites and muscle chemoreflex activation (Osada et al. 2015; Tanaka et al. 2014). Furthermore, the concurrent cooling of the skin increases sympathetic activation and vascular resistance (Johnson et al. 2014; Charkoudian 2010). Their combination can further increase cardiac workload among healthy persons and CAD patients (Manou-Stathopoulou et al. 2015). During whole-body superficial cooling, the decrease in T_sk_ persisted throughout the graded exercise in this thermal condition (4 °C decrease in T_sk_ by the end of exposure).

Differing from dynamic exercise, we observed a lower HR when individuals exercised in the cold, with this lower HR being sustained at all exercise intensities. Despite a reduced HR, the magnitude of its response toward bouts of graded static exercise remained the same at both environmental temperatures (Fig. 2C). The observed bradycardic response towards isometric exercise in the cold is consistent with prior findings among healthy persons (Mäkinen et al. 2008). Static exercise itself is known to augment both sympathetic and vagal activity (González-Camarena et al. 2000). It is further possible that the increased vagal activity related to facial cold exposure (approximately 15 °C decrease in facial skin temperature) stimulates the trigeminal nerve and evokes a non-baroreflex mediated vagal response (Khurana and Wu 2006) that reduce HR further when compared to normothermic exercise. Such a response apparently is maintained during the rest cycles for static (Fig. 2C), but not for dynamic exercise (Fig. 1C).

To our knowledge, there are only a few studies that have examined cardiovascular responses related to upper-body static exercise in the cold. Those studies employed the isometric handgrip test (3 min at 30% of maximal voluntary contraction) and involved only healthy subjects (Greaney et al. 2014; Koutnik et al. 2014; Mäkinen et al. 2008). Consistent with the findings of our study, Mäkinen et al. (2008) demonstrated higher brachial and systolic BPs, and lowered HR, when the isometric handgrip exercise was performed at + 10 °C (whole-body cold exposure) compared with + 25 °C. Koutnik et al. (2014) detected a higher aortic BP during isometric handgrip exercise in the cold (+ 4 °C), but unaltered brachial SBP, DBP and HR compared with exercise at + 20 °C. Greaney et al. (2014) found no effects of concomitant whole-body (head-out) cooling and isometric exercise either on muscle sympathetic nerve activity (MSNA) or BP. These somewhat deviating findings could be due to differences in study populations, as well as duration and, intensity and forms of cold exposure and exercise. Nevertheless, we describe for the first time how cardiovascular responses of CAD patients are affected during graded upper-body (bench press) static exercise in combination with whole-body cold exposure.

### Electrocardiogram

Against our hypothesis, we did not find markedly altered ECG responses during upper-body dynamic or static exercise in the cold. We assumed that the higher cardiac workload could result in a mismatch between myocardial oxygen demand and supply among CAD patients whose myocardial blood flow is limited (Manou-Staphopoulou et al. 2015). Such an occurrence would be manifest as earlier and more profound appearance of myocardial ischemia (ST-segment depression exceeding 1 mm) while exercising in the cold, which has been shown among CAD patients during symptom-limited maximal ergometer exercise in a cold (− 20 °C) environment (Meyer et al. 2010). We only detected ST depression exceeding 1 mm during upper-body dynamic exercise at the highest exercise intensity that was, however, not related to temperature. Earlier studies related to cold exposure, involving a considerable decrease in body temperature, have detected ECG changes at rest (Aslam et al. 2006). On the other hand, changes in cardiac repolarization at rest may also occur with superficial cooling (Hintsala et al. 2014). In our study, most of the ECG parameters were not affected by exercise or temperature. Only QT interval, which is known to be strongly dependent on HR (Andersen et al. 2008), shortened during moderate and heavy dynamic upper-body exercise in cold compared with a neutral environment. However, this apparent QT-interval shortening was no longer observed when these responses were corrected for HR.

### Applicability

These results allow for an understanding of how chores performed with the upper body, such as snow shoveling or chopping of wood, affect cardiovascular functions in a cold environment. This information is relevant, as previous studies have showed a higher incidence of cardiac events, such as myocardial infarctions in the cold season (Liu et al. 2015; Fares 2011). These events often involve persons having ischemic heart disease (Toukola et al. 2015; Janardhan et al. 2010) and who are engaged with sudden heavy exercise, such as shoveling or pushing snow (Smolander et al. 1995; Sheldahl et al. 1992) which involves elements of both dynamic and static exercise. In contrast, our study showed that sustained submaximal upper-body exercise in the cold increases cardiovascular strain but does not alter ECG parameters among stable asymptomatic CAD patients. This observation provides support for this population to continue to follow the recommended exercise guidelines, even in the cold, supporting their cardiac rehabilitation (Anderson et al. 2016). The provided information can be useful for health care professionals and rehabilitation experts in advising their clients of the healthy and safe of wintertime exercise to promote cardiac health. The expected benefits for the patients include maintaining and improving their functional capacity and working ability also during the cold season.

### Strengths and limitations

The strengths of the study include strictly controlled level of thermal exposure and exercise. Furthermore, each subject served as his own control; therefore, eliminating potential confounders due to interindividual factors. In addition, randomization of the trials limits an order effect. Finally, strict selection of participants helps reduce confounding variables from causes other than those related to cardiovascular diseases. Restricting enrollment to only those with stable CAD precludes us from distinguishing the observed responses from other disease states or healthy persons. For safety reasons, we did not cease medication of the patients during the experiments. Hence, we evaluated cardiovascular responses of individuals who are being treated for CAD, rather than examining the disease in the absence of medical treatment.

## Conclusions

Our results show that sustained submaximal upper-body dynamic and static exercise in a cold environment increases cardiovasculars strain (blood pressure and RPP during dynamic exercise) in stable CAD patients but does not markedly alter their cardiac electrical function. Further studies are suggested that consider the role of disease severity, comorbidity and medication related to CAD.

## Data Availability

The present data are available on request from the authors.

## Abbreviations

BP: Blood pressure
CAD: Coronary artery disease
ECG: Electrocardiogram
HR: Heart rate
RPE: Rate of perceived exertion
MAP: Mean arterial pressure
MVC: Mean voluntary contraction
RPP: Rate pressure product
Tsk: Mean skin temperature
